# Managing Multivalvular Bioprosthetic Failure and a Hidden Aortic Root–Left Ventricular Outflow Tract Fistula With a Transcatheter Approach: Balancing Risk and Intervention

**DOI:** 10.1016/j.cjco.2024.12.006

**Published:** 2024-12-18

**Authors:** Julius Jelisejevas, Ali Husain, Brian Chiang, Howard Paje, Hassan Ogran, Desiree Nadine Wussler, Stephanie L. Sellers, Jonathon A. Leipsic, Philipp Blanke, Richard Cook, Janarthanan Sathananthan, John G. Webb, David A. Wood

**Affiliations:** a Centre for Cardiovascular Innovation, St Paul’s and Vancouver General Hospital, Vancouver, British Columbia, Canada; b Centre for Heart Valve Innovation, St Paul’s Hospital, University of British Columbia, Vancouver, British Columbia, British Columbia, Canada; c University of British Columbia, Vancouver, British Columbia, Canada; d Sheikh Jaber Al-Ahmad Hospital, Kuwait; e Cardiovascular Translational Laboratory, Providence Research Centre for Heart Lung Innovation, Vancouver, British Columbia, Canada

Managing multiple failed bioprosthetic heart valves in high-risk patients poses challenges in the lifelong management of valvular heart disease. Transcatheter options offer a less invasive and often the only viable option in this patient cohort.^1^ Here, we report a case of a young patient who initially underwent surgical multivalvular replacement at the age of 33 years secondary to congenital rubella syndrome.

The 41-year-old man, who uses a wheelchair, with a small body size (154 cm, 37 kg) and a complex cardiac history, necessitating surgical aortic and mitral valve replacement with, respectively, 23-mm and 27-mm Carpentier-Edwards Perimount Magna Ease valves (Edwards Lifesciences) as well as tricuspid valve annuloplasty, was admitted to the hospital with refractory heart failure due to multivalve failure 8 years after the initial intervention. Transthoracic echocardiography documented a failed surgical aortic valve with severe transvalvular regurgitation and severe bioprosthetic mitral stenosis (mean gradient 24 mm Hg). Computed tomography indicated a low risk for coronary obstruction with aortic valve-in-valve (VIV) implantation, with small-calibre (4-5 mm) noncalcified iliofemoral arteries and a significant anticipated risk of neo–left ventricular outflow tract (LVOT) obstruction with transseptal mitral VIV replacement (TS-MVIVR) (Fig. 1E). His surgical risk was deemed to be prohibitive.

**Figure 1:**
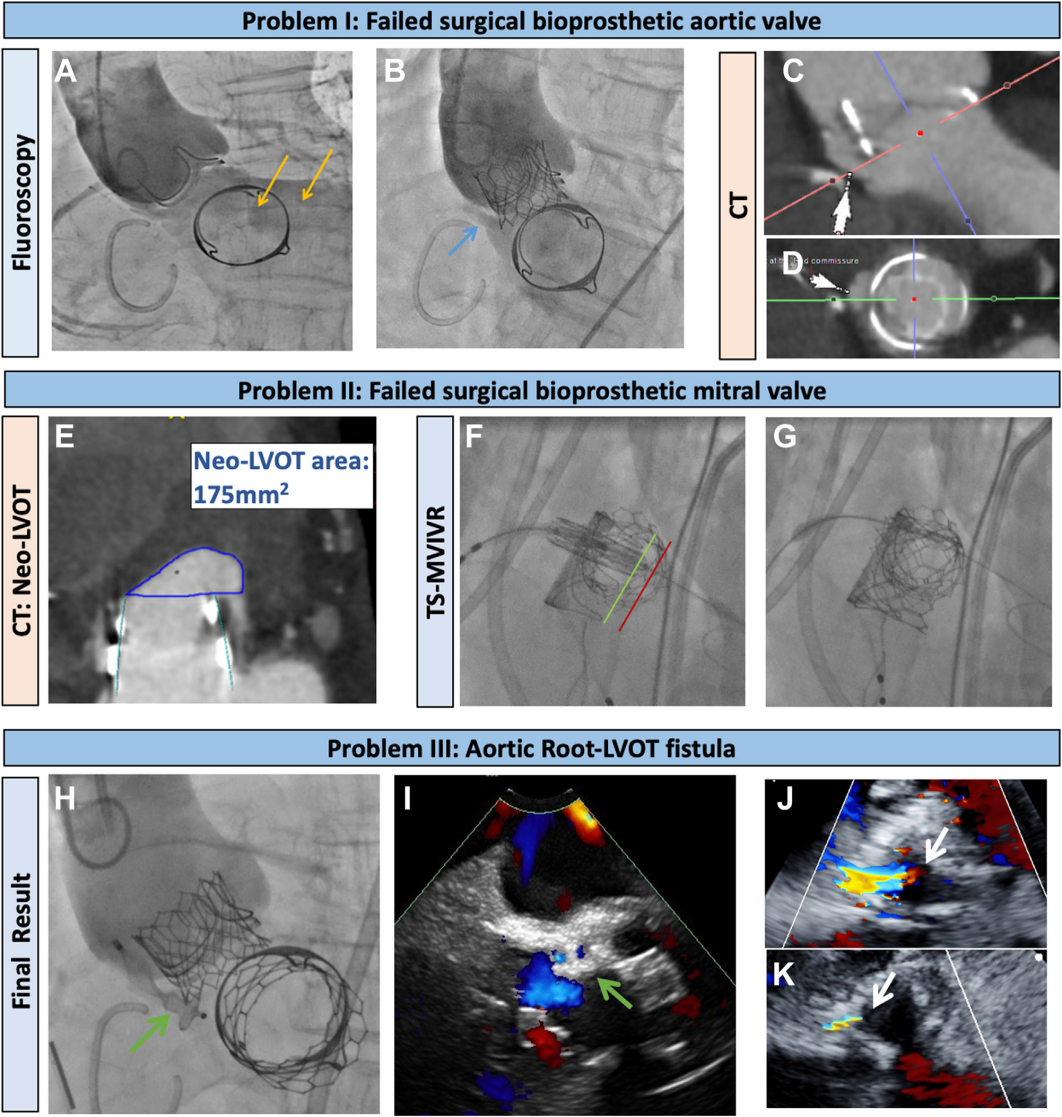
Managing multivalvular failure and hidden aortic root–LVOT fistula. (**A**) Aortic root angiogram pre-VIV indicates severe regurgitation (yellow arrows). (**B**) Post-VIV demasks communication (blue arrow) between aortic root and LV, which was obscured by dominant transvalvular regurgitation. (**C, D**) Pre VIV computed tomography (CT) revealed dehiscence at the level of sewing ring of bioprosthetic aortic valve at non- and right coronary commissure (white arrows). (**E**) High anticipated risk of Neo-Left-Ventricular-Outflow-Tract (Neo-LVOT) obstruction on CT. (**F, G**) Ventricular portion of THV (green line) positioned in line with the stent posts of surgical bioprosthesis to minimize risk of Neo-LVOT obstruction with a good final result. (**H, I**) Final aortic root angiogram after paravalvular leak closure under fluoroscopic and ICE guidance showing good position of the device (green arrows) with significant reduction of paravalvular regurgitation. (**J, K**) TTE pre- and post- paravalvular leak closure with a 10 × 5 mm Amplatzer Valvular Plug III device confirming significant reduction in paravalvular regurgitation (white arrows). ICE, intracardiac echocardiogram; LV, left ventricle; THV, transcatheter heart valve; TS-MVIVR, transseptal mitral valve in valve replacement; TTE, transthoracic echocardiogram; VIV, valve in valve.

After heart team discussion, a 23-mm Sapien Ultra valve (Edwards Lifesciences) was used via transfemoral approach for aortic VIV replacement, which proceeded uneventfully. However, postprocedure aortic root angiography revealed an aortic root–LVOT fistula with significant regurgitation, initially underappreciated owing to dominant transvalvular regurgitation (Video 1; Fig. 1, A and B). This was later confirmed on review of the pre-VIV CT, which revealed dehiscence, which had been initially overlooked. Plans were made to address this electively in combination with TS-MVIVR, but the patient was readmitted 3 weeks later secondary to congestive heart failure accelerated by Covid-19 pneumonia.

After reassessment, it was decided to proceed with the intervention. Attempts to insert even a paediatric transesophageal echocardiography probe were unsuccessful, resulting in TS-MVIVR and fistula closure under conscious sedation, with intracardiac echocardiography (ICE) used as an alternative in addition to fluoroscopy. After an ICE-guided transseptal puncture, the ventricular side of a 26-mm Sapien 3 Ultra transcatheter heart valve (Edwards Lifesciences) was positioned in line with the posts of the surgical bioprosthetic, rather than in the usual “deeper” position of the valve, to mitigate the risk of the neo-LVOT obstruction, achieving a good final result (Fig. 1, F and G). This was followed by the successful retrograde closure of the aortic root–LVOT fistula with the use of a 10 × 5 mm Amplatzer Vascular Plug III (Abbott) under ICE and fluoroscopy guidance (Fig. 1, F-K). The combined procedure was well tolerated, and the patient was discharged after 1 week with good outcomes at the 3-month follow-up.

This case underscores the complexity and challenges of multimodal imaging and the management of failed bioprosthetic heart valves in high-risk patients with limited surgical options. Underlying pathologies, such as paravalvular fistulas, may be obscured and overlooked in the presence of dominant transvalvular regurgitation and require careful attention in periprocedural planning to optimise outcomes in this patient population.

## Supplementary Material

Video 1 Aortic root angiography before TAV-in-SAV indicates severe transvalvular regurgitation, obscuring the presence of paravalvular regurgitation; post–TAV-in-SAV angiography reveals communication between aortic root and left ventricle with significant regurgitation. SAV, surgical aortic valve; TAV, transcather aortic valve.
